# Temperature curve of raw human milk heated by different methods: experimental study

**DOI:** 10.1590/1980-220X-REEUSP-2023-0130en

**Published:** 2024-01-26

**Authors:** Ana Cristina Freitas de Vilhena Abrão, Gisele de Jesus Schmidt, Maria José Guardia Mattar, Carla Santos Cruz, Juliana de Barros Barbosa, Dariza Zimiani Daré, Kelly Pereira Coca

**Affiliations:** 1 Universidade Federal de São Paulo, Escola Paulista de Enfermagem, Departamento Enfermagem na Saúde da Mulher, São Paulo, SP, Brazil.; 2 Universidade Federal de São Paulo, Escola Paulista de Enfermagem, São Paulo, SP, Brazil.; 3 Secretaria de Estado da Saúde de São Paulo, SP, Brazil.

**Keywords:** Human milk, Human milk banks, Neonatal intensive care unit, Prematurity, Leche humana, Bancos de leche humana, Unidad de cuidados intensivos neonatales, Prematuridad, Leite humano, Bancos de leite humano, Unidade de terapia intensiva neonatal, Prematuridade

## Abstract

**Objective:**

To analyze the temperature curve of raw or pasteurized human milk exposed to different heating methods.

**Method:**

Experiments with volumes of 5 ml to 100 ml of human milk were carried out between 2016 and 2021 and analyzed according to the exposure time by different heating methods. Descriptive statistics included the calculation of means, medians, minimum and maximum values, measures of dispersion and standard deviation.

**Results:**

The thermal curve made it possible to identify the heating of human milk close to body temperature when subjected to a water bath and microwaves. Milk exposed to room temperature (21°C) was unable to reach this temperature. When heated in a water bath at 40°C, smaller volumes reached body temperature between 3 and 5 minutes, while in a microwave at 50% power, practically all volumes reached temperature.

**Conclusion:**

The temperature curves of raw or pasteurized human milk were constructed, and it was possible to verify its behavior using different heating methods for administering the food in a neonatal intensive care unit, considering the volume, type and time of heating and temperature.

## RESUMO

**Objetivo:**

Analisar a curva de temperatura do leite humano cru ou pasteurizado exposto a diferentes métodos de aquecimento.

**Método:**

Experimentos com volumes de 5 ml a 100 ml de leite humano foram realizados entre 2016 e 2021 e analisados segundo o tempo de exposição por diferentes métodos de aquecimento. A estatística descritiva incluiu o cálculo das médias, medianas, valores mínimos e máximos, medidas de dispersão e desvio padrão.

**Resultados:**

A curva térmica permitiu identificar o aquecimento do leite humano próximo da temperatura corporal quando submetidos a banho-maria e micro-ondas. O leite exposto à temperatura ambiente (21°C) não foi capaz de atingir tal temperatura. No aquecimento em banho-maria a 40°C, volumes menores alcançaram a temperatura corporal entre 3 e 5 minutos, enquanto em micro-ondas na potência de 50%, praticamente todos os volumes alcançaram essa temperatura.

**Conclusão:**

As curvas de temperatura do leite humano cru ou pasteurizado foram construídas, sendo possível verificar o seu comportamento mediante diferentes métodos de aquecimento para administração do alimento em unidade de terapia intensiva neonatal, considerando o volume, tipo e tempo de aquecimento e temperatura.

## RESUMEN

**Objetivo:**

Analizar la curva de temperatura de la leche humana cruda o pasteurizada expuesta a diferentes métodos de calentamiento.

**Método:**

Se realizaron experimentos con volúmenes de 5 ml a 100 ml de leche humana entre 2016 y 2021 y se analizaron en función del tiempo de exposición mediante diferentes métodos de calentamiento. La estadística descriptiva incluyó el cálculo de medias, medianas, valores mínimos y máximos, medidas de dispersión y desviación estándar.

**Resultados:**

La curva térmica permitió identificar el calentamiento de la leche humana próximo a la temperatura corporal cuando se sometió a baño maría y microondas. La leche expuesta a temperatura ambiente (21°C) fue incapaz de alcanzar esta temperatura. Cuando se calentó en un baño de agua a 40°C, los volúmenes más pequeños alcanzaron la temperatura corporal entre 3 y 5 minutos, mientras que en un microondas al 50% de potencia, prácticamente todos los volúmenes alcanzaron la temperatura.

**Conclusión:**

Se construyeron las curvas de temperatura de la leche humana cruda o pasteurizada y se pudo comprobar su comportamiento utilizando diferentes métodos de calentamiento para administrar el alimento en una unidad de cuidados intensivos neonatales, teniendo en cuenta el volumen, el tipo y el tiempo de calentamiento y la temperatura.

## INTRODUCTION

The benefits for children’s health of exclusive breastfeeding are widely recognized in the literature^(1,2)^. The World Health Organization (WHO) recommends starting breastfeeding within the first hour of the neonate’s life, practicing exclusive breastfeeding (EBF) until six months of age and, in a complementary way with other safe foods, continuing with breastfeeding until two years of age or more^(3)^. Breastfeeding is the ideal way to provide children with the nutrients they need for healthy growth and development, acting to prevent obesity and infectious diseases until adolescence, as well as improving intelligence levels^(4)^.

Similarly, for preterm newborns (PTNB), human milk (HM) continues to be the nutrition of choice due to its short- and long-term benefits^(5)^, such as lower incidence of necrotizing enterocolitis, sepsis and chronic lung diseases^(6)^; as well as being better tolerated due to its easy digestibility and high nutritional quality, and also promoting bonding between the binomial^(7,8)^.

For these reasons, the use of pasteurized donor HM is the food of first choice in the absence of the mother’s own milk^(9)^. Thus, Human Milk Banks (HMB) have emerged aiming to meet this demand as well as guaranteeing food and nutritional security for newborns^(10,11)^.

One of the challenges for feeding infants who do not get their HM directly from their mother’s breast is the ideal temperature at which it should be given to infants in the neonatal intensive care unit (NICU). When HM is collected, it should be stored frozen at minus 3°C^(12)^ and after pasteurization at minus 4°C^(13)^. When extracted at the Human Milk Collection Point (HMCP) to be offered from mother to child, it must be refrigerated at 5°C^(14)^. Pasteurized HM is thawed in a water bath at 40°C to be portioned into volumes for the next 24 hours, according to the prescription^(15)^.

There are no established evidences or recommendations for methods of warming human milk for administration to hospitalized children in Brazil. The only recommendation in this regard is a technical standard issued by the Brazilian Network of Human Milk Banks, which states that aliquots of refrigerated milked HM should be heated in a water bath at approximately 36°C^(16)^, without considering other heating methods, or the volume, container and time ratio. In this sense, a study carried out in the state of São Paulo sought to find out about the practices adopted in relation to the heating of human milk by the HMBs of the São Paulo Network^(17)^. Of the 92 HMBs and HMCPs in the state of São Paulo in 2016, 39 (42.3%) answered the questionnaire. Of these, 66% carried out heating, the majority using a water bath, without specifying the length of time it was used or the temperature of the device. As for the importance of warming up, 74.3% believed that warming up milk was important in order to avoid giving cold milk and to ensure better acceptance by the newborn and the approximation of the child’s body temperature. On the other hand, those who didn’t heat the milk were concerned about the loss of nutritional properties and the difficulty in knowing the heating time due to the variations in volumes offered.

Offering HM close to body temperature (36°C to 37.5°C)^(18)^ can prevent food intolerance among premature infants, contributing to the child’s proper growth and development^(19,20)^.

In view of the above, this study aimed to analyze the temperature curve of raw or pasteurized human milk exposed to different heating methods.

## METHOD

### Type of Study

Three experiments were carried out with samples of human milk involving measuring the temperature of the milk when exposed to room temperature (RT), heated in a water bath (WB) and in a microwave oven (MO).

### Location

The experiments were carried out from 2016 to 2021 at the Human Milk Collection Station of the São Paulo Hospital of the Federal University of São Paulo (HSP/UNIFESP), a unit linked to the Ana Abrão Center for Breastfeeding Incentive and Support/Human Milk Bank of UNIFESP.

### Samples

The samples consisted of aliquots of donated HM considered to be unsuitable for consumption, after quality assessment in the pasteurization process, for being outside the quality standards and which were frozen at –20°C, separated specifically for the experiments. A total of 1088 samples were used, 480 for the room temperature experiment, 360 for the water bath and 248 for the microwave, giving a total volume of 45,440 ml of human milk.

### Data Collection

Data was collected in 2016, 2017 and 2021. The samples were separated with the volumes established using a 20 ml disposable syringe luer slip without needle, and placed in containers commonly used for distributing HM in clinical practice in Brazil (80 ml polypropylene dosing cup; 120 ml polypropylene bottle and 20 ml syringe). A penetration thermometer (digital spit type, waterproof –45+230°C, Incoterm, calibration certificate with Inmetro/RBC traceability) was used to measure the temperature of the human milk samples used in the experiment. A digital stopwatch was used to control the time of the experiments.

The experiments were carried out in the following sequence:

*Sample preparation*: HM samples were removed from the freezer and thawed in a water bath (model ALTS-102E- EME equipment) previously heated to 40°C until cooled liquid milk was obtained^(15)^. The samples were then placed on reusable ice on an aluminum stand to be portioned into the volumes defined for the experiments. To do this, a 20 ml syringe was used to obtain the desired volume and transferred to the specific container, exposed at room temperature (21°C). The portioned samples were then stored in stainless steel trays in the fridge at 4°C for two hours. The containers used to store the samples were a 20 ml syringe, a polypropylene measuring cup for volumes up to 60 ml and a polypropylene bottle for larger volumes.

*Temperature measurement:* the sample was taken out of the fridge and placed on an aluminum stand, the lid was removed from the container in the case of a measuring cup or bottle, the sample was homogenized with a brief circular movement, and the outlet temperature (T0) was measured with a penetration thermometer in a perpendicular position and centered on the lower third of the cup. The final temperature was read once it had stabilized. If the syringe was used as a container, the contents were placed in a measuring cup so that the temperature could be measured.

The experiments consisted of exposing the HM samples to three heating methods: room temperature (average 21°C), a water bath at 40°C and a 20-liter microwave at 50% power.

*Experiment 1*: four different samples were tested for each volume of HM. The volumes of 5 ml, 10 ml, 15 ml and 20 ml placed in a measuring cup were exposed to room temperature for times determined in minutes (5, 10, 15, 20, 25 and 30). For volumes of 30 ml, 40 ml, 50 ml and 60 ml in a measuring cup and volumes of 70 ml, 80 ml, 90 ml and 100 ml in a polypropylene bottle, exposure took place in minutes (5, 10, 15, 20, 25, 30, 35, 40, 45, 50, 55 and 60). In total, there were 480 HM samples.

*Experiment 2*: four different samples of each volume of HM (5 ml, 10 ml, 15 ml, 20 ml, 30 ml, 40 ml, 50 ml and 60 ml) packed in a measuring cup and 90 ml in a polypropylene bottle were heated simultaneously for times in minutes (1, 2, 3, 4, 5, 10, 15, 20, 25 and 30), totaling 360 samples of HM. In both experiments, the level of water in the equipment depended on the volume, as the condition established was that the container should remain immobile, without floating or being submerged, so the cups or flasks were of the same size, shape and volume. The volume of water was placed above the volume of HM, but without reaching or overlapping the lid of the container, as recommended by the Brazilian Network of Human Milk Banks^(15)^.

*Experiment 3*: four different samples of each volume of HM (10 ml, 15 ml and 20 ml) were placed in a measuring cup and replicated equally in a syringe, heated for times in seconds (2, 5, 8, 10, 15 and 20). The 25 ml and 30 ml volumes, also placed in a measuring cup, were heated for the same times as described above. For the 60 ml volume, placed in a measuring cup, and the 90 ml volume, placed in a polypropylene bottle, heating took place in seconds (2, 5, 8, 10, 15, 20 and 25). The total number of HM samples tested was 248. All the samples were placed in the center of the microwave dish.

Before the experiments were carried out, the samples were refrigerated for approximately two hours and the temperature checks were carried out once for each time on all four samples, to obtain an average temperature for each volume/time, used to construct the temperature curve.

After checking, the temperatures were recorded on a specific form and the human milk samples were discarded.

### Data Analysis and Processing

The data obtained was stored in an Excel spreadsheet for Mac version 16.43. For descriptive statistical analysis, means, medians and minimum and maximum values were calculated, and the standard deviation was used to measure dispersion. The time versus volume ratio was analyzed according to the heating method to which the milk was exposed. The analyses were repeated four times and the average value obtained was considered in the curve.

### Ethical Aspects

The study was compliant with the guidelines and norms No. 466/2012 of the National Health Council for research with biological samples. The project was cleared by the Research Ethics Committee of the Federal University of São Paulo under numbers 1.372.931(17/12/2015); 1.903.777 (01/02/2017); n. 2.362.044(1/11/2017); and n. 4.411.874(20/11/2020).

## RESULTS

**Figure 1:**
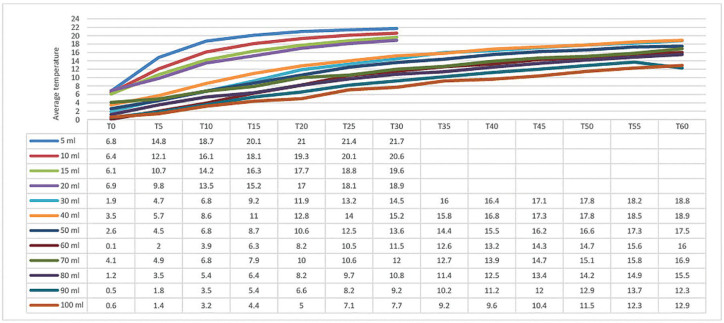
Average temperatures obtained for all the volumes analyzed in relation to the time spent at room temperature.

**Figure 2:**
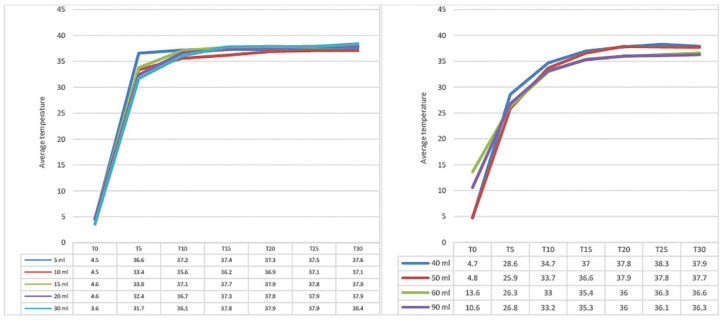
Average temperature obtained for volumes of 5 ml to 30 ml analyzed in relation to the time spent heating in the water bath. São Paulo, 2022.

**Figure 3:**
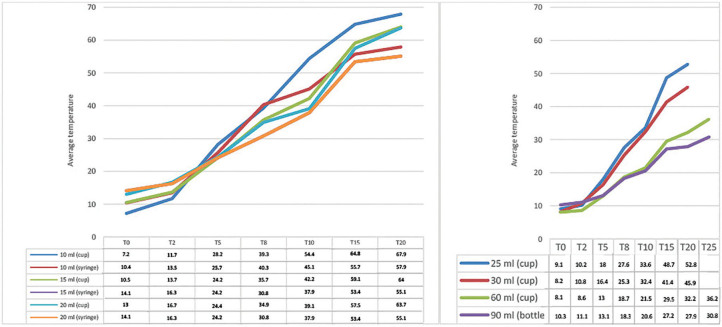
Average temperatures obtained for all the volumes analyzed in relation to the time taken for microwave heating. São Paulo, 2022.

The means, medians, minimum and maximum values and standard deviations were calculated for each volume tested according to the heating method; however, only the mean was used to draw up the temperature curves, the results of which are shown in Figures 1 to 3.

With regard to HM exposed to room temperature, the results showed that none of the volumes tested were close to body temperature at 60 minutes (Figure 1).

In experiment 2, with heating in a water bath, it was found that for the 5 ml volume, body temperature was reached in 5 minutes, for the 10 ml to 30 ml volumes in 10 minutes, and for the other volumes in 20 minutes. After this period, all the volumes showed a tendency for the temperature to stabilize (Figure 2).

In experiment 3, heated with microwave at 50% power, the results showed that for the volumes packed in syringes, in the volume of 10 ml, there was a large variation in temperature between 5 and 8 seconds of heating (27.7°C–40.9°C), exceeding body temperature. For the 15 ml volume, values reached those very close to body temperature after 8 seconds of heating (36.2°C). For 20 ml, body temperature was reached between 8 and 10 seconds (30.8°C–37.9 °C). The results obtained for the other volumes in cup and bottle containers can be seen in Figure 3.

Table 1 shows a summary of the results obtained by heating the HM according to the experiments carried out.

**Table 1:** Summary of the results obtained by heating HM, according to volume, container and time considering body temperature – São Paulo, SP, Brazil, 2023.

Container	Volumes (ml)	Heating time water bath at 40°C (min)	Heating time – microwave (50% power) (seconds)
**Dosing cup**	05	5	–
	10	10	8
	15–20	15	8
	25	–	10
	30	15	10
	40–50	20	–
	60	20	25
**Syringe**	10–20	–	8
**Bottle**	90	20	–

## DISCUSSION

The thermal curve allowed to identify human milk heated up to close to newborn’s body temperature when subjected to both water baths and microwaves. Milk exposed to room temperature (21°C) was unable to reach this temperature. This is the first study to propose heating human milk based on human milk thermal curve experiments.

A randomized clinical trial carried out on premature babies with a birth weight under 1500 grams or a gestational age of less than 34 weeks, which aimed to examine the effects of administering lukewarm milk compared to milk at room temperature, found that warming milk close to body temperature was preferable to room temperature, since the newborns showed a reduction in residual gastric amount, apnea of prematurity and the need for treatment for reflux^(21)^. The recommendation to give milk at body temperature has also been emphasized, since breastfeeding performed directly on the breast has values within this temperature range, and administration at room temperature causes a reduction in the temperature of the premature newborn^(22)^. In view of these findings, it is important to highlight the importance of these results to support practices in the care of newborns in neonatal ICUs, especially premature infants, given the lack of consensus among HMBs in Brazil.

With regard to heating, the results show that using water bath or microwave for heating are both viable methods. The Centers for Disease Control and Prevention^(23)^ and The Academy of Breastfeeding Medicine^(24)^ recommend that thawed human milk kept at room temperature should be administered within a maximum of two hours, since the shorter the exposure time, the better the milk quality. In this sense, exposure at room temperature was not effective because none of the volumes tested reached body temperature within 60 minutes, and there was a tendency for the temperature to stabilize after this period. The results show that it is not feasible to consider this method because, according to the prescriptions of the babies’ diets, the interval between them usually varies between 1 and 3 hours.

On the other hand, heating in a water bath at 40°C could be a method to meet the most recent technical standard of the Brazilian Network of Human Milk Banks^(16)^, which stated the need for heating, and advises that aliquots of refrigerated human milk should be heated in a water bath at approximately 36°C at the times predetermined in the prescription, without, however, considering the issue of volume and heating time. Therefore, the findings of this study corroborate current recommendations, supporting the practice of heating human milk in a water bath.

The results showed that, for small volumes, the temperature can reach values above body temperature in short periods of time, which can cause the milk to suffer changes in its unique bioactive properties. One study showed that breast milk, like other foods, is sensitive to temperature variations and can undergo changes in some nutrients and in its bioactive properties, for example in relation to the denaturation of enzymes, leading to their subsequent loss of biological function. During heating, not only the temperature the milk can reach, but also the length of time it is exposed to heat, are important for enzymatic activity^(25)^.

In water bath heating, the main difficulty was maintaining the water level in the equipment, since it is not possible to add different volumes of milk at the same time. Therefore, for each volume to be analyzed, the volume of water in the water bath must be adapted to preserve the vertical position of the containers and avoid contaminating the milk with the water in the water bath. In this sense, the water bath method makes it impossible to heat the milk in a syringe-type container.

Although little discussed in Brazil, another method of heating human milk could be microwaving. It was found that for all volumes except the largest (90 ml) it was possible to reach temperatures similar to body temperature within the proposed time. However, this technique requires greater care, since the temperature can rise rapidly, especially for the smallest volumes, leading to a loss of milk quality, as well as presenting a risk factor for scalding. The microwave provides volumetric heating, although it is not uniform and for this reason overheating can be lower^(26)^. Among the recommendations for use are precise control of the appliance’s power, the need to mix the milk to ensure temperature uniformity, and direct monitoring of the temperature in order to achieve safe heating^(26)^.

There are debates in the literature regarding the use of microwaves to heat human milk. Studies from the 1980s and 1990s indicated acceleration of *E. coli* and degradation of IgA and lysozyme when milk was heated in domestic microwaves^(27,28)^. In contrast, recent studies have not reported similar negative effects. The content of nutrients and fatty acids did not change after heating milk in microwaves, confirming that using the device under controlled conditions can be a promising pasteurization method^(26,29)^. Furthermore, the use of microwaves appears to be beneficial, as identified in a study that tested samples of human milk contaminated with cytomegalovirus that were heated in a high-powered commercial microwave for 30 seconds and showed 100% efficacy in eradicating the virus, while low-powered microwaves did not achieve complete neutralization, with a failure rate of 13%^(30)^.

It is therefore understood that both water bath and microwave heating can be used in the neonatal ICU routine, as they require less time to reach body temperature. However, in order to guarantee quality, it is extremely important to respect the containers in which the milk will be stored and the volume/time ratio. In microwave heating, the use of glass containers would be ideal, given that heating in polypropylene containers is questionable, according to a recently published study^(31)^.

In this sense, the summary of the results shown in Table 1 could be used as a suggestion for heating human milk close to body temperature of 36°C to 37°C, according to volume, container and time.

The main difficulties in carrying out the study were obtaining the amount of human milk needed to carry out the experiments; matching the level of the water in the water bath with the level of the milk in the containers so that they were all in an upright position; and the limited literature on the subject.

## CONCLUSION

By analyzing the thermal curves of human milk exposed to different heating methods, it was possible to provide a parameter for professionals working in HMBs for the preparation of human milk for administration in neonatal ICUs, considering the triad of volume, type and time of heating and temperature.

Future studies could explore the thermal control of the children who received the food and the quality of the human milk subjected to the recommended temperatures.

## References

[1] Rollins NC, Bhandari N, Hajeebhoy N, Horton S, Lutter CK, Martines JC (2016). Why invest, and what it will take to improve breastfeeding
practices?. Lancet..

[2] Silprasert A, Dejsarai W, Keawvichit R, Amatayakul K (1987). Effect of storage on the creamatocrit and total energy content of
human milk.. Hum Nutr Clin Nutr..

[3] World Health Organization. (2003). Global strategy for infant and young child feeding [Internet]..

[4] Santiago ACT, Cunha L, Vieira NSA, Oliveira Moreira LM, Oliveira PR, Lyra PPR (2019). Breastfeeding in children born small for gestational age and
future nutritional and metabolic outcomes: a systematic
review.. J Pediatr (Rio J)..

[5] Ballard O, Morrow AL (2013). Human milk composition: nutrients and bioactive
factors.. Pediatr Clin North Am..

[6] Parker MG, Stellwagen LM, Noble L, Kim JH, Poindexter BB, Puopolo KM (2021). Promoting human milk and breastfeeding for the very low birth
weight infant.. Pediatrics..

[7] Luna E, Parkar SG, Kirmiz N, Hartel S, Hearn E, Hossine M (2022). Utilization efficiency of human milk oligosaccharides by
human-associated akkermansia is strain dependent.. Appl Environ Microbiol..

[8] Demers-Mathieu V, Qu Y, Underwood MA, Borghese R, Dallas DC (2018). Premature infants have lower gastric digestion capacity for human
milk proteins than term infants.. J Pediatr Gastroenterol Nutr..

[9] Abrams SA, Landers S, Noble LM, Poindexter BB (2017). Donor human milk for the high-risk infant: preparation, safety,
and usage options in the United States.. Pediatrics..

[10] Agência Nacional de Vigilância Sanitária. (2008). Bancos de leite humano: funcionamento, prevenção e controle de riscos
[Internet]..

[11] Brasil, Ministério da Saúde. (2017). Bases para discussão da política nacional de promoção, proteção e apoio
ao aleitamento materno [Internet]..

[12] Rede Global de Bancos de Leite Humano. (2021). Norma técnica 18.21 [Internet]..

[13] Rede Global de Bancos de Leite Humano. (2021). Norma técnica 36.21 [Internet]..

[14] Rede Global de Bancos de Leite Humano. (2021). Norma técnica 22.21 [Internet]..

[15] Rede Global de Bancos de Leite Humano. (2021). Norma técnica 24.21 [Internet]..

[16] Rede Global de Bancos de Leite Humano. (2018). Norma técnica 47.18 [Internet]..

[17] Silva AM (2021). A cobertura dos bancos de leite humano e postos de coleta de leite
humano da região metropolitana de São Paulo de acordo com as redes regionais
de atenção à saúde [dissertação]..

[18] Potter P, Perry AG (2018). Fundamentos de enfermagem..

[19] Gonzales I, Duryea EJ, Vasquez E, Geraghty N (1995). Effect of enteral feeding temperature on feeding tolerance in
preterm infants.. Neonatal Netw..

[20] Çamur Z, Erdoğan Ç (2023). The effect of breast milk temperature on feeding intolerance in
tube-fed preterm infants: a randomized controlled study.. J Neonatal Nurs..

[21] Uygur O, Yalaz M, Can N, Koroglu OA, Kultursay N (2019). Preterm infants may better tolerate feeds at temperatures closer
to freshly expressed breast milk: a randomized controlled
trial.. Breastfeed Med..

[22] Lawlor-Klean P, Lefaiver CA, Wiesbrock J (2013). Nurses’ perception of milk temperature at delivery compared to
actual practice in the neonatal intensive care unit.. Adv Neonatal Care..

[23] Center for Disease Control and Prevention. (2022). Human milk storage guidelines [Internet]..

[24] Eglash A, Simon L (2017). ABM Clinical protocol #8: human milk storage information for home
use for full-term infants, revised 2017.. Breastfeed Med..

[25] Bransburg-Zabary S, Virozub A, Mimouni FB (2015). Human milk warming temperatures using a simulation of currently
available storage and warming methods.. PLoS One..

[26] Levchenko A, Lukoyanova O, Borovik T, Levchenko M, Sevostianov D, Sadchikov P (2017). The novel technique of microwave heating of infant formulas and
human milk with direct temperature monitoring.. J Biol Regul Homeost Agents..

[27] Quan R, Yang C, Rubinstein S, Lewiston NJ, Sunshine P, Stevenson DK (1992). Effects of microwave radiation on anti-infective factors in human
milk.. Pediatrics..

[28] Carbonare SB, Palmeira P, Silva ML, Carneiro-Sampaio MM (1996). Effect of microwave radiation, pasteurization and lyophilization
on the ability of human milk to inhibit *Escherichia coli*
adherence to HEp-2 cells.. J Diarrhoeal Dis Res..

[29] Martysiak-Żurowska D, Malinowska-Panczyk E, Orzolek M, Kusznierewicz B, Kielbratowska B (2022). Effect of microwave and convection heating on selected nutrients
of human milk.. Food Chem..

[30] Ben-Shoshan M, Mandel D, Lubetzky R, Dollberg S, Mimouni FB (2016). Eradication of cytomegalovirus from human milk by microwave
irradiation: a pilot study.. Breastfeed Med..

[31] Hussain KA, Romanova S, Okur I, Zhang D, Kuebler J, Huang X (2023). Assessing the release of microplastics and nanoplastics from
plastic containers and reusable food pouches: implications for human
health.. Environ Sci Technol..

